# Sexual function of overweight pregnant women with gestational diabetes mellitus: A cross‐sectional study

**DOI:** 10.1002/hsr2.70080

**Published:** 2024-09-18

**Authors:** Saloumeh Peivandi, Ali Habibi, Seyed H. Hosseini, Mohammad Khademloo, Elham Motamedi‐Rad

**Affiliations:** ^1^ Department of Obstetrics and Gynecology, Faculty of Medicine, Clinical Research Development Unit of Imam Khomeini Hospital Mazandaran University of Medical Sciences Sari Iran; ^2^ Student Research Committee, Faculty of Medicine Mazandaran University of Medical Sciences Sari Iran; ^3^ Psychiatry and Behavioral Sciences Research Center Mazandaran University of Medical Sciences Sari Iran; ^4^ Department of Community Medicine, School of Medicine Mazandaran University of Medical Sciences Sari Iran

**Keywords:** gestational diabetes, overweight, physiological sexual dysfunction, psychological sexual dysfunction, pregnancy

## Abstract

**Background and Aims:**

Overweight and obesity are on the rise worldwide and may affect female sexual function. The aim of this study was to investigate the relationship between sexual function in normal and overweight pregnant women with gestational diabetes mellitus (GDM).

**Methods:**

This cross‐sectional study was conducted in overweight and normal‐weight pregnant women with GDM in Sari, Iran. Data were collected from 2018 to 2021. The demographic data collected from the participants included age, educational level, occupation, gestational age, duration of marriage, number of births, place of residence (city or village), private house, private bedroom, and insurance status. The General Health Questionnaire, Female Sexual Function Index (FSFI), and Enriched Marital Satisfaction questionnaires were used to assess mental health, sexual functioning, and marital satisfaction, respectively.

**Results:**

The study included 200 women with GDM. The mean age of the participants was 29.75 (SD = 4.40) years. Among pregnant women with GDM, 56.50% of them had sexual dysfunction based on FSFI. The mean FSFI score in pregnant women with GDM was 25.60 (SD = 3.61). Among the participants, 50.00% had normal body mass index (BMI). There was no significant difference between BMI status and total sexual function score (*p* > 0.05). The multivariate analysis revealed that marital satisfaction (β = 0.41, *p* < 0.001) and BMI status (*β* = −0.15, *p* = 0.002) were the only factors significantly associated with overall sexual function, explaining 27% of the variance in the total FSFI score.

**Conclusion:**

The results of this study showed that there was no significant relationship between sexual dysfunction and obesity in GDM. Considering that the research in this area is very limited and the negative effects of obesity and GDM have been confirmed in many areas, including sexual desire disorder, necessary planning should be done to control these factors.

## 1 INTRODUCTION

Sexuality is a vital aspect of overall health and well‐being, influenced by multiple factors such as biology, psychology, society, culture, and religion.^1^ Sexual dysfunction, a common disorder in both men and women, receives particular attention in sexual health. However, female sexual dysfunction often goes unnoticed. A precise definition of sexual dysfunction is crucial for estimating its prevalence, determining its causes, and identifying appropriate interventions, including drug treatments.^2^ Sexual dysfunction refers to a range of problems that prevent individuals from engaging in desired sexual relationships. Studies indicate that sexual dysfunction affects 40%–50% of women, with rates increasing with age from 15% to 33% in young individuals to 40%–50% in those over 60 years old.^3^, ^4^ In Iran, a meta‐analysis found a prevalence of female sexual dysfunction at approximately 43.9%.^5^

Pregnancy is a transformative period for women, characterized by physical, hormonal, and psychological changes that significantly impact sexual health.^6^ Throughout each trimester, specific changes affect sexual behavior. Many women experience reduced sexual desire and satisfaction as their pregnancy progresses, influenced by physical and psychological changes, as well as social, cultural, and religious factors. Concerns about potential harm to the fetus also play a role.^6^, ^7^ The third trimester can particularly impair various aspects of the female sexual response cycle. Studies report sexual dysfunctions in 31%–85% of pregnant women, with the highest occurrence in the third trimester.^4^, ^8^, ^9^ Surprisingly, 75% of pregnant women lack knowledge about sexual intercourse during pregnancy and are hesitant to discuss it during prenatal visits. This lack of information can contribute to sexual dysfunction.^10^

Gestational diabetes mellitus (GDM) refers to glucose intolerance during pregnancy and is the most common endocrine disorder in pregnant women. Approximately 7% of pregnancies involve diabetes, with prevalence rates ranging from 1% to 14% based on diagnostic criteria and demographics.^11^ GDM prevalence is expected to rise globally.^12^ In Iran, GDM prevalence varies across regions, averaging at 4.9%.^13^ Individuals with chronic type 1 or type 2 diabetes often experience peripheral neuropathy or vascular lesions that can affect sexual function.^14^, ^15^ Hyperglycemia is associated with increased serum prolactin levels and altered neurotransmitter activity, potentially contributing to sexual dysfunction.^16^, ^17^ Although women with GDM are not at high risk for sexual dysfunction due to the acute onset of the condition, changes in neurohormonal levels during GDM may lead to metabolic alterations resulting in sexual dysfunction. For instance, metabolic issues like obesity can negatively impact sexual performance. Some studies have shown a significant association between GDM and sexual function in women,^18^, ^19^ while others have produced mixed results, finding no correlation.^17^

Obesity and overweight, defined as the excessive accumulation of fat, pose health risks. Obesity is characterized by a body mass index (BMI) greater than 30, while overweight falls between 25 and 29.9 on the BMI scale.^20^ Both conditions can affect the sexual performance of women of reproductive age.^20^, ^21^, ^22^, ^23^, ^24^ Obese or overweight pregnant women face a higher risk of depression and anxiety, leading to lower quality of life and an increased likelihood of experiencing sexual dysfunction symptoms.^22^ Limited research exists on the relationship between sexual dysfunction, GDM, and overweight. Therefore, this study aims to investigate the relationship between sexual dysfunction in pregnant women with GDM and those with a higher BMI than normal.

## 2 METHODS

This cross‐sectional study was conducted in Sari, northern Iran, from 2018 to 2021, and focused on overweight and normal weight pregnant women with GDM. Inclusion criteria for participants included being pregnant women aged 18–40 years with GDM, having a singleton pregnancy, and having a body mass index (BMI) between 18.5 and 40. Exclusion criteria consisted of multiple pregnancies, ectopic pregnancy, premature rupture of the amniotic sac, cervical insufficiency, premature birth, type 1 or type 2 diabetes, ongoing treatment for vaginal infection, drug addiction, alcoholism, pregnancy resulting from sexual assault, recent hospitalization within the past month, or absence of sexual activity due to the absence of a husband. The sample size for this study was determined based on a calculation considering a worldwide prevalence of 60% for sexual dysfunction and 40% for obesity. At a level of *α* = 0.05 with a power of 0.8, the sample size was determined to be 200.

### 2.1 Ethical considerations

The research protocol was approved by the ethics committee of Mazandaran University of Medical Sciences, Iran (code: IR.MAZUMS.IMAMHOSPITAL.REC.1398.052). Written informed consent was obtained from all participants before their participation in the study. Participants were informed about the study's purpose, the confidentiality of the data, and how the data would be used for research purposes only.

### 2.2 Data collection

Women who tested positive for diabetes screening between the 24th and 28th week of pregnancy were assigned to one of two groups, each consisting of 100 participants. The groups were categorized based on their pre‐pregnancy BMI: those with a normal BMI (18.5–24.9) and those with a high BMI ( ≥ 25). Demographic information was collected from all participants, including age, education level, occupation, gestational age, duration of marriage, number of previous births, place of residence (city or village), type of housing (private house or other), and insurance status. The mental health of the participants was evaluated using the General Health Questionnaire (GHQ‐12), their sexual function was assessed using the Female Sexual Function Index (FSFI), and their marital satisfaction was measured using the Enriched Marital Satisfaction (EMS) questionnaire.

### 2.3 Questionnaire

#### 2.3.1 GHQ‐12

The GHQ‐12 is a self‐report questionnaire used to track people with a mental disorder. This questionnaire can be considered as a set of questions that consists of the lowest levels of common symptoms of illness that exist in various mental disorders, and thus it can identify mental patients as a general class of those who consider themselves healthy. GHQ‐12 consists of 6 positive items and 6 negative items. The lower the score, the more mental health a person has, and a score higher than 12 is considered a mental disorder.^25^ The validity and reliability of this questionnaire in Iran was conducted by Montazeri et al.^26^ and Cronbach's *α* was 0.87.

#### 2.3.2 FSFI

The FSFI is a six‐dimensional instrument that measures women's sexual performance with 19 questions in the dimensions of libido, sexual arousal, vaginal smoothness or wetness, orgasm, sexual satisfaction, and pain. The scores considered for each question are 1–5 points for sexual desire and 0–5 points for sexual arousal, vaginal lubrication, orgasm, sexual satisfaction, and pain. The individual's score in each section is added to the scores of the questions related to that section and by multiplying the score of each section by its coefficient (coefficient of sexual desire = 0.6, sexual arousal = 0.3, vaginal moist = 0.3, orgasm = 0.3, sexual satisfaction = 0.4, and sexual pain = 0.4) is calculated. If the person's score is less than 26.55, it indicates sexual dysfunction, and the higher the score, the better the sexual performance. Also, the cutoff points for the subscales of libido, arousal, sexual lubricity, orgasm, satisfaction, and pain are considered to be 3.3, 3.4, 3.4, 3.4, 3.8, and 3.8, respectively.^27^ The validity and reliability of this questionnaire in Iran were conducted by Fakhri et al.^28^

#### 2.3.3 EMS

The EMS consists of 115 closed questions and 12 subscales, except for the first subscale which has five questions, the rest of the subscales have 10 questions. The answer to the questions is in the form of five options (strongly agree—agree—neither agree nor disagree—disagree—strongly disagree).^29^ The validity and reliability of this questionnaire were conducted in Iran by Arab Alidousti et al.^30^ and the Cronbach's *α* coefficient was 0.76.^30^

### 2.4 Statistical analysis

Data were analyzed using the SPSS software package (version 16.0, SPSS Inc.). Quantitative variables are described by mean and standard deviation, and qualitative variables are described by number (percentage). The *t*‐test and chi‐squared tests were used to examine the relationship between variables. To examine the factors associated with sexual function in this population of overweight pregnant women with GDM, a series of multivariate linear regression analyses were conducted. The total FSFI score and each of the FSFI subscale scores (desire, arousal, lubrication, orgasm, satisfaction, and pain) were modeled as the dependent variables in separate regression analyses. The independent variables included in the regression models were age, number of births, marriage duration, pregnancy weeks, education level, occupation, residence, home ownership, insurance status, BMI status, and marital satisfaction. For statistical significance, two‐sided *p*‐values less than 0.05 were considered statistically significant.

## 3 RESULTS

### 3.1 Participants

As shown in Table 1, 200 women with GDM were included in this study. The mean age of the participants was 29.75 (SD = 4.40) years. The mean number of births for these women was 0.59 (SD = 0.75). The mean duration of their marriage was 4.70 (SD = 3.46) years. The mean number of weeks of pregnancy was 28.09 (SD = 3.46). Among the participants, 49.50% had a university degree and 92.50% were housewives. Also, 55.5% of them lived in the city, 75.50% of them had their own house and 75.50% of them had their own bedroom. Among the participants, 96.50% had insurance.

**Table 1:** Patient characteristics and sexual function based on FSFI (*N* = 200).

	Total (*N* = 200)	FSFI		*p*‐Value
		With sexual dysfunction (*N* = 113)	Without sexual dysfunction (*N* = 87)	
Age		29.30 (SD = 4.41)	30.20 (SD = 4.40)	0.181*
Number of births		0.60 (SD = 0.73)	0.59 (SD = 0.78)	0.642*
Duration of marriage		4.50 (SD = 3.89)	4.90 (SD = 3.04)	0.632*
Gestational age		27.80 (SD = 2.96)	28.39 (SD = 3.16)	0.346*
Level of education				
High school	50 (25.00)	39 (78.00)	11 (22.00)	0.000**
Diploma	99 (49.50)	62 (62.63)	37 (37.37)	
University education	51 (25.50)	12 (23.53)	39 (76.47)	
Occupation				
Housewife	185 (92.50)	112 (60.54)	73 (39.46)	0.000**
Employed	15 (7.50)	1 (6.67)	14 (93.33)	
Place of residence				
City	111 (55.50)	47 (42.34)	64 (57.66)	0.000**
Village	89 (44.50)	66 (74.16)	23 (25.84)	
Private house				
Yes	151 (75.50)	79 (52.32)	72 (47.68)	0.046**
No	49 (24.50)	34 (69.39)	15 (30.61)	
Private bedroom				
Yes	151 (75.50)	100 (66.23)	82 (33.77)	0.214**
No	49 (24.50)	13 (26.53)	36 (73.47)	
Insurance				
Yes	193 (96.50)	108 (55.96)	85 (44.04)	0.701**
No	7 (3.50)	5 (71.43)	2 (28.57)	
Supplementary insurance				
Yes	19 (9.50)	3 (15.97)	16 (84.21)	0.000**
No	181 (90.50)	110 (60.77)	71 (39.23)	

*Note*: Values are given as a number (percentage) for categorical variables and mean (standard deviation) for continuous variables.

Abbreviation: FSFI, Female Sexual Function Index.

* *p*‐Value was obtained with an independent *t*‐test

** *p*‐Value was obtained with a chi‐square test.

### 3.2 Patient characteristics and sexual function based on FSFI

As shown in Table 1, independent *t*‐test and chi‐squared test were used to analyze the difference between the means and frequencies of patient characteristics and sexual function. Among pregnant women with GDM, 56.50% of them had sexual dysfunction based on FSFI. There was a significant difference between sexual function based on FSFI and level of education (*p* < 0.001), occupation (*p* < 0.001), place of residence (*p* < 0.001), private house (*p* = 0.046), and supplementary insurance (*p* < 0.001).

### 3.3 Sexual function based on FSFI and BMI status

As shown in Table 2, chi‐squared test was used to analyze the difference between the means and frequencies of sexual function and BMI status. The mean score of FSFI in pregnant women with GDM was 25.60 (SD = 3.61). There was no significant difference between BMI status and total sexual function score based on FSFI (*p* = 0.254). However, there was a significant difference between BMI status and the sexual desire subscale (*p* = 0.009).

**Table 2:** Sexual function based on FSFI and BMI status (*N* = 200).

	Total (*N* = 200)	BMI		*p*‐Value
		Normal (*N* = 100)	Above normal (*N* = 100)	
FSFI subscales				
Sexual desire		3.73 (SD = 0.97)	3.78 (SD = 0.66)	
With dysfunction	42 (21.00)	29 (69.50)	13 (30.50)	0.009
Without dysfunction	158 (79.00)	71 (44.94)	87 (55.06)	
Sexual arousal		3.75 (SD = 0.86)	3.79 (SD = 0.95)	
With dysfunction	56 (28.00)	27 (48.21)	29 (51.79)	0.875
Without dysfunction	144 (72.00)	73 (50.69)	71 (49.31)	
Vaginal lubrication		4.18 (SD = 0.87)	4.33 (SD = 0.83)	
With dysfunction	33 (16.50)	15 (45.45)	18 (54.55)	0.704
Without dysfunction	167 (83.50)	85 (50.90)	82 (49.10)	
Orgasm		4.44 (SD = 1.03)	4.33 (SD = 0.95)	
With dysfunction	32 (16.00)	15 (46.88)	17 (53.12)	0.700
Without dysfunction	168 (84.00)	85 (50.60)	83 (49.40)	
Sexual satisfaction		4.66 (SD = 0.94)	4.84 (SD = 0.79)	
With dysfunction	40 (20.00)	25 (62.50)	15 (37.50)	0.077
Without dysfunction	160 (80.00)	75 (46.88)	85 (53.12)	
Sexual pain		4.59 (SD = 0.76)	4.78 (SD = 0.74)	
With dysfunction	27 (13.50)	15 (55.56)	12 (44.44)	0.535
Without dysfunction	173 (86.50)	85 (49.13)	88 (50.87)	
FSFI total		25.37 (SD = 3.62)	25.83 (SD = 3.60)	
With sexual dysfunction	113 (56.50)	61 (53.98)	52 (46.02)	0.254
Without sexual dysfunction	87 (43.50)	39 (44.83)	48 (55.17)	

*Note*: Values are given as a number (percentage) for categorical variables and mean (standard deviation) for continuous variables. *p*‐Value was obtained with a chi‐square test.

Abbreviations: BMI, body mass index; FSFI, Female Sexual Function Index.

The frequency of sexual dysfunction was 64% in the second trimester and 49.5% in the third trimester, which was statistically significant (*p* = 0.036). Among the subscales of sexual function, sexual desire, and vaginal moisture had a significant relationship with BMI in the second trimester (*p* = 0.001 and *p* = 0.022, respectively). However, in the third trimester of pregnancy, the only subscale that had a significant relationship with BMI was vaginal moisture (*p* = 0.003). Comparing the total score of the FSFI questionnaire and each of its subscales with the level of marital satisfaction obtained from the EMS questionnaire shows that the total score of sexual function had a significant relationship with marital satisfaction (*p* < 0.001). Thus, people with higher levels of marital satisfaction (high satisfaction and very high satisfaction) have more normal sexual function. Also, the subgroups of sexual arousal and sexual satisfaction have a significant relationship with the level of marital satisfaction (*p* = 0.026 and *p* < 0.001, respectively).

The multivariate analysis for the total FSFI score revealed that marital satisfaction (*β* = 0.41, *t* = 8.76, *p* < 0.001) and BMI status (*β* = −0.15, *t* = −3.15, *p* = 0.002) were the only factors significantly associated with overall sexual function. The regression model explained 27% of the variance in the total FSFI score (*R*‐squared = 0.27). When analyzing the FSFI subscales, marital satisfaction emerged as the primary factor significantly associated with the desire (*β* = 0.37, *t* = 7.80, *p* < 0.001), arousal (*β* = 0.39, *t* = 8.23, *p* < 0.001), lubrication (*β* = 0.42, *t* = 9.03, *p* < 0.001), orgasm (*β* = 0.38, *t* = 8.01, *p* < 0.001), satisfaction (*β* = 0.44, *t* = 9.55, *p* < 0.001), and pain (*β* = −0.35, *t* = −7.32, *p* < 0.001) domains. BMI status was also significantly associated with the desire (*β* = −0.14, *t* = −2.94, *p* = 0.003) and total FSFI (*β* = −0.15, *t* = −3.15, *p* = 0.002) subscales.

## 4 DISCUSSION

This study found that 56.5% of pregnant women with GDM experienced sexual dysfunction. Similar studies in Brazil reported rates of 51.7% and 66.7%.^19^, ^31^ An Ethiopian study involving 398 pregnant women with GDM found a frequency of 53.3%.^32^ In a meta‐analysis study conducted in Turkey, Ugurlu et al. concluded that differences in the prevalence of sexual dysfunction across studies were not solely attributed to variations in study quality, but could also be influenced by differences in demographic characteristics, as well as social and cultural factors among the participants.^33^ An Iranian study reported a frequency of 87.3% for sexual dysfunction.^34^ The present study had a lower prevalence due to the exclusion of individuals with potential mental disorders. Education level, occupation, place of residence, supplementary insurance, and homeownership correlated with sexual dysfunction, while variables like age, number of births, length of marriage, week of pregnancy, having a private bedroom and basic insurance did not. These factors can influence the quality of life and sexual performance of pregnant women. Another study found no significant association between sexual performance and demographic factors in women with GDM.^35^

Several studies have explored the impact of GDM on sexual dysfunction, either independent of BMI or considering BMI in non‐GDM individuals.^17^, ^18^, ^19^, ^34^, ^35^, ^36^, ^37^, ^38^ However, research specifically examining the effect of BMI on sexual dysfunction in individuals with GDM is limited.^31^ Findings from these studies have been contradictory, with some showing a significant association between GDM and sexual dysfunction,^18^, ^19^, ^36^ while others find no significant association.^17^, ^35^ In this study, no relationship was found between sexual dysfunction and BMI, but there was a significant difference in the sexual desire subscale, indicating varied sexual desire levels based on BMI. This differs from a study that found an association between sexual dysfunction in pregnant women with GDM and BMI.^31^ Additionally, sexual desire and stimulation scored lowest among the sexual function subscales in this study, consistent with previous research suggesting that sexual desire is more affected by diabetes and pregnancy than other aspects of sexual functioning. Other studies involving pregnant women have also reported lower sexual performance scores in the sexual desire and arousal subscale for women with GDM.^19^ Decreased sexual desire and increased sexual dysfunction during pregnancy have been consistent findings in most studies involving pregnant women.^17^, ^18^, ^19^, ^38^

This study shows that marital satisfaction influences the sexual function of pregnant women with GDM. Higher marital satisfaction correlates with more normal sexual function, particularly in the areas of arousal and satisfaction. A study by Tabandeh et al.^35^ found no significant difference in marital satisfaction or sexual performance between women with GDM and healthy pregnant women.

This study has several limitations that should be considered when interpreting the results. First, the cross‐sectional design of the study limits the ability to infer causal relationships between the examined factors and sexual function. Additionally, the self‐reported nature of the data collected through questionnaires may be subject to social desirability bias, where participants may underreport or over report certain behaviors or experiences. Moreover, the study was conducted in a single center, which may limit the generalizability of the findings to other populations. Future studies should consider a longitudinal design and utilize objective measures, such as physiological assessments, in addition to self‐reported data to provide a more comprehensive understanding of the factors influencing sexual function in this population. Additionally, evaluating sexual function in the same pregnant women across different trimesters would provide valuable insights into how sexual function changes throughout pregnancy.

## 5 CONCLUSION

The results of this study showed that there was no significant relationship between sexual dysfunction and obesity in GDM. Considering that the research in this area is very limited and the negative effects of obesity and GDM have been confirmed in many areas, including sexual desire disorder, necessary planning should be done to control these factors.

## AUTHOR CONTRIBUTIONS

**Saloumeh Peivandi**: Conceptualization; funding acquisition; writing—original draft; writing—review and editing; methodology; supervision; project administration; validation; investigation. **Ali Habibi**: Conceptualization; writing—original draft; writing—review and editing; resources; data curation; validation; visualization; methodology; project administration. **Seyed Hamzeh Hosseini**: Conceptualization; writing—review and editing; validation; methodology; resources. **Mohammad Khademloo**: Formal analysis; methodology; validation; visualization; writing—original draft; software. **Elham Motamedi‐Rad**: Data curation; resources; writing—review and editing; methodology; conceptualization.

## CONFLICT OF INTEREST STATEMENT

The Mazandaran University of Medical Sciences, which provided support for this study, was not involved in the study design, data collection, analysis, and interpretation, the writing of the report, or the decision to submit the report for publication. The remaining authors declare no conflict of interest.

## TRANSPARENCY STATEMENT

The lead author Ali Habibi affirms that this manuscript is an honest, accurate, and transparent account of the study being reported; that no important aspects of the study have been omitted; and that any discrepancies from the study as planned (and, if relevant, registered) have been explained.

## Data Availability

The data that support the findings of this study are available on request from the corresponding author. The data are not publicly available due to privacy or ethical restrictions.
